# Cases of Mediterranean spotted fever in southeast of Iran

**Published:** 2020-06

**Authors:** Mehrdad Farrokhnia, Zohreh Yousefi Ghalejoogh, Mahdi Rohani, Ahmad Ghasemi, Saber Esmaeili, Ehsan Mostafavi

**Affiliations:** 1 Infectious and Tropical Research Center, Kerman University of Medical Sciences, Kerman, Iran; 2 Department of Epidemiology and Biostatistics, Research Centre for Emerging and Reemerging Infectious Diseases, Pasteur Institute of Iran, Tehran, Iran; 3 Department of Microbiology, Pasteur Institute of Iran, Tehran, Iran; 4 National Reference Laboratory for Plague, Tularemia and Q Fever, Research Centre for Emerging and Reemerging Infectious Diseases, Pasteur Institute of Iran, Akanlu, Kabudar Ahang, Hamadan, Iran

**Keywords:** Mediterranean spotted fever, *Rickettsia conorii*, Tache noire, Tick-borne disease

## Abstract

In this study the clinical manifestations, laboratory findings, and management of five patients diagnosed with Mediterranean spotted fever (MSF) from southeast of Iran are presented. All patients but one had recent tick-bite histories which were noticeable as black eschars (tache noire). Patients’ samples were tested by real-time PCR and serology (IFA). The disease was confirmed by fourfold rising of IgG antibodies against *Rickettsia conorii*. This is the first report of MSF cases in Iran.

## INTRODUCTION

Mediterranean spotted fever (MSF) is an acute febrile illness. *Rickettsia conorii*, the causative agent of MSF is maintained in nature through a vertebrate-arthropod cycle. Humans are accidental hosts of this bacterium (1). MSF is characterized by fever, flu-like symptoms, headache, rash, and a distinct mark which is known as tache noire at the site of the bite (2). This black eschar is pathognomonic but is not seen in all cases (3). These symptoms along with serology constitute the diagnosis basis of MSF (2).

*Rhipicephalus sanguinus*, the vector and reservoir of this infection, has a worldwide distribution (4); Kerman in southeast of Iran with hot climate is no exception (5). Domestic dogs are *Rh. Sanguineus’s* main hosts. Some factors like warm temperature increase this tick’s attack to humans (6). No licensed vaccine is available for preventing spotted fever group of rickettsial infections. Antibiotic should not be prescribed as prophylaxis. However, treatment with antibiotic must be started in any suspected MSF cases before the diagnosis confirmation (7).

*R. conorii* is distributed in Europe, Africa, and Asia (8). The only study investigating the prevalence of *R. conorii* in Iran goes back to 1996. Seropositive response against *R. conorii* was detected in eleven out of 40 (27.5%) of human sera examined in that study (9). In some other middle east countries like Oman, the first reports of this disease were returned to 1990s but further studies have been shown that this zoonosis disease is endemic in rural regions of this country (10).

There is a lack of seroepidemiological data in Iran. Thus, an estimation of the prevalence of MSF and its public health impact in Iran is not clear.

Here we present the clinical manifestations, laboratory findings, and management of five patients diagnosed with MSF from rural areas of two cities in the southeast of Iran.

## CASE HISTORY

**Fig. 1:**
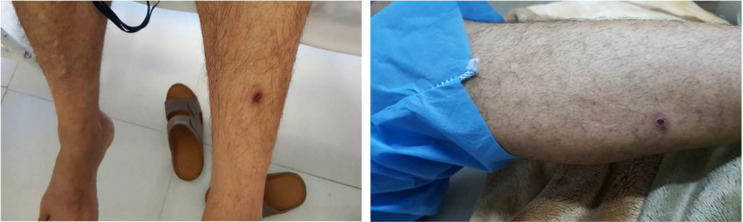
Cutaneous manifestation of Mediterranean spotted fever patients in southeast of Iran, 2017–2018; Tache noir (a dark plaque overlying a shallow ulcer) in two of the patients.

Between May 2017 and February 2018, five patients, including four men who worked in livestock farms with the mean age of 46, and a two-year-old girl were admitted to Afzalipour hospital in Kerman province, Iran (Table 1). All patients were from rural areas of Zarand and Kahnuj counties, Kerman. All patients were first misdiagnosed, but following their symptoms getting worse they were all transferred to the capital city of Kerman province. They all had about a week history of varying degrees of fever, headache, myalgia, chill, diarrhea, anorexia and diffuse maculopapular rash on their abdomens and limbs at the time of hospitalization (Table 2). None of them had apparent bleeding. However, all patients but one had recent tick-bite history which were noticeable as black eschars (tache noire) with about 10 millimeters in diameter (Fig. 1). These necrotic ulcers were observed in the abdominal walls and the legs. The cases were first misdiag-nosed as poisoning in one case, Kawasaki disease in one other case and suspected cases of CCHF for the rest.

**Table 1.** Epidemiological feature of Mediterranean spotted fever patients in southeast of Iran, 2017–2018

**Epidemiological feature**	**Case 1**	**Case 2**	**Case 3**	**Case 4**	**Case 5**
Gender	male	male	male	male	Female
Age	55	51	45	33	2
Job	Livestock farmer	Livestock farmer	Livestock farmer	Livestock farmer	-
Contact with ticks	Yes	Yes	Yes	Yes	No
City	Zarand	Zarand	Kahnuj	Kahnuj	Kahnuj
Time before admission (days)^¥^	8	3	7	7	7
Hospitalization Period (days)	7	8	7	5	8
Initial diagnosis	CCHF	CCHF	Poisoning	CCHF	Kawasaki Disease

¥ Time spent with symptoms before hospital admission (days)

**Table 2.** Clinical features of Mediterranean spotted fever patients in southeast of Iran, 2017–2018

**Clinical features**	**Case 1**	**Case 2**	**Case 3**	**Case 4**	**Case 5**
Fever	+	+	+	+	+
Rash	+	+	+	+	+
Headache	+	+	+	+	−
Myalgia	+	+	+	+	−
Diarrhea	−	−	−	+	+
Chills	+	+	+	+	+
Nausea and Vomiting	+	−	+	−	+
Anorexia	+	+	+	+	+

Regarding hematological parameters, thrombocytopenia and elevated levels of serum aspartate aminotransferase (AST) and alanine transferase (ALT) were found in all five patients (>50 U/L, median for ALT, 91U/L and for AST, 115 U/L). Lactate Dehydrogenase (LDH) level was abnormal in three patients (60%) (>190 U/L, median 662 U/L) and in two patients (40%) prothrombin time (PT) (>13.5, median 19.5) and Internationalized Normalized Ratio (INR) was high (>1.1, median 1.3). WBC count, bilirubin (BIL), and partial thromboplastin time (PTT) were normal in all patients (Table 3). Not fulfilling CCHF criteria and regarding the clinical manifestations and laboratory results mentioned above, the patients were suspected of having MSF. DNA was extracted from all sera samples and tested for 16S rRNA gene of *Rickettsia* spp. by real-time PCR (11). Sera samples were tested by IFA (Vircell, Spain) for detection of antibodies against *R. conorii*. Molecular results of all samples were negative for *Rickettsia* spp. All five patients were confirmed by rising of IgG antibodies against *R. conorii* (Table 3). Three patients had fourfold rising of IgG antibodies titers (patients NO. 1, 2 and 5). Also, patients number 3 and 4 had more than fourfold rise in IgG titers (16-fold and 8-fold, respectively).

**Table 3.** Laboratory investigations of Mediterranean spotted fever patients in southeast of Iran, 2017–2018

**Blood investigations**	**Case 1**	**Case 2**	**Case 3**	**Case 4**	**Case 5**
White cell count (×10^9^/L)	4500	5200	4400	4900	11800
Platelet count (×10^9^/L)	88000	81000	81000	116000	30000
Hemoglobin (g/dl)	14.9	20.1	12	15.1	9.1
Alanine aminotransferase (U/L)	91	381	112	68	83
Aspartate aminotransferase (U/L)	113	475	121	77	115
Bilirubin	Normal	Normal	Normal	Normal	Normal
Lactate dehydrogenase (U/L)	-	1143	662	-	918
Creatine phosphokinase (U/L)	-	185	98	-	-
Alkaline phosphatase (U/L)	2.3	3.9	176	250	478
Prothrombin time	12.5	13	25	12	14
International normalized ratio	1	1	1.3	1	1.3
Partial thromboplastin time	38	38	39	38	39
Titers of IgG^*^ in fist serum sample	1:128	1:512	1:128	1:64	1:128
Titers of IgG^*^ in second serum sample	1:512	1:2048	1:2048	1:512	1:512

* by IFA.

Doxycycline was administered to all patients (100 mg every 12 hours for the adults and 5 mg/kg for the child). Antibiotic therapy was well tolerated and effective and improvement in all patients was significant. Recovery was achieved in less than a week with no observed clinical sequel.

## DISCUSSION

This is the first report of Mediterranean spotted fever (MSF) in Iran. The initial diagnosis of MSF was based on clinical grounds which were later confirmed by serology. As it was expected, the molecular tests in all of these samples were negative, which may be due to the delay in sample collection for molecular tests. According to MSF guidelines, since antibiotic therapy may affect the accuracy of molecular and culture-based methods, samples are recommended to be taken before the initiation of antibiotic treatment (12). In this study the MSF diagnosis was confirmed by IgM seroconversion or a four-fold increase of IgG antibody titers in paired sera. In all of the patients, fever, rashes and anorexia were observed and the level of liver transaminases was elevated. Although these patients were first diagnosed as suspected cases of CCHF, the clinical manifestations and the improvement of symptoms after antibiotic treatment (doxycycline) was suggestive of MSF.

Thick-borne zoonoses are a public health concern throughout the world, including Iran. In the last few decades the number of human rickettsioses has increased (8). The reasons abound, of which improved diagnostic tools, and socio-political changes like cross border movements and climate change can be mentioned. On the other hand, spotted fever group (SFG) rickettsial infection including MSF may have been under–documented in previous years. Lack of knowledge and poor diagnostic methods are among the most important reasons of ITS misdiagnosis (13).

It is recommended that more studies and a surveillance system on knowing the situation of rickettsial diseases in Iran be conducted. MSF should be differentiated from other tick-borne zoonoses which have sudden flu-like symptoms like SFG rickettsioses, CCHF, and Lyme disease etc. CCHF is the most significant tick-borne viral human infectionin Iran (14). In each year, almost 100 to 150 cases of this disease are reported in Iran. However, only about 25% of probable cases of CCHF are reported as confirmed cases of this disease. Since the geographic distribution of MSF human cases encompasses Middle East, Indian subcontinent, and Turkey besides Europe (Mediterranean basin) and Africa (15, 16), the investigation of MSF in the rest 75% of patients, especially in those with tick bite history and rash is recommended to be conducted.

*R. conorii* have four subspecies based on epidemiological and clinical differences: *R. conorii* subsp. *conorii*, *R. conorii* subsp. *israelensis*, *R. conorii* subsp. *caspia* and *R. conorii* subsp. *indica*, all geographically restricted to the Eastern Hemisphere. In Turkey (the northwestern neighbor), MSF is an endemic diseases and several PCR and serological confirmed clinical cases were annually reported from this country (17).

In Iran, many physicians are still unaware of MSF. Guidelines on the diagnosis and treatment must be provided and health care providers should be notified of MSF diagnostic and therapeutic considerations. The probable cases of MSF should be reported. Studies on investigating the prevalence of the causative agent, vector, and reservoir of this rickettsial infection are needed to be carried out and the presence of *Rickettsia* spp. in ticks should be studied as well.
